# Nuclei Detection for 3D Microscopy With a Fully Convolutional Regression Network

**DOI:** 10.1109/ACCESS.2021.3073894

**Published:** 2021-04-19

**Authors:** MARYSE LAPIERRE-LANDRY, ZEXUAN LIU, SHAN LING, MAHDI BAYAT, DAVID L. WILSON, MICHAEL W. JENKINS

**Affiliations:** 1 Department of Biomedical Engineering, Case Western Reserve University, Cleveland, OH 44106, USA; 2 Department of Electrical Engineering and Computer Science, Case Western Reserve University, Cleveland, OH 44106, USA; 3 Department of Radiology, Case Western Reserve University, Cleveland, OH 44106, USA; 4 Department of Pediatrics, Case Western Reserve University, Cleveland, OH 44106, USA

**Keywords:** 3D microscopy, cell detection, cell segmentation, centroid detection, deep learning, regression, spatial statistics, V-net, whole tissue

## Abstract

Advances in three-dimensional microscopy and tissue clearing are enabling whole-organ imaging with single-cell resolution. Fast and reliable image processing tools are needed to analyze the resulting image volumes, including automated cell detection, cell counting and cell analytics. Deep learning approaches have shown promising results in two- and three-dimensional nuclei detection tasks, however detecting overlapping or non-spherical nuclei of different sizes and shapes in the presence of a blurring point spread function remains challenging and often leads to incorrect nuclei merging and splitting. Here we present a new regression-based fully convolutional network that located a thousand nuclei centroids with high accuracy in under a minute when combined with V-net, a popular three-dimensional semantic-segmentation architecture. High nuclei detection F1-scores of 95.3% and 92.5% were obtained in two different whole quail embryonic hearts, a tissue type difficult to segment because of its high cell density, and heterogeneous and elliptical nuclei. Similar high scores were obtained in the mouse brain stem, demonstrating that this approach is highly transferable to nuclei of different shapes and intensities. Finally, spatial statistics were performed on the resulting centroids. The spatial distribution of nuclei obtained by our approach most resembles the spatial distribution of manually identified nuclei, indicating that this approach could serve in future spatial analyses of cell organization.

## I. INTRODUCTION

The explosion of new tissue clearing protocols in the last decade has revolutionized the field of three-dimensional (3D) microscopy [1]. It is now possible to image intact organs, developing embryos and even whole mice [2]–[5]. Imaging tissue samples without sectioning preserves tissue integrity, cell shape and organization, and provides a more complete view of the patterns and connections that enables organs to function. Whole-organ imaging with single-cell resolution is bringing us toward organism-level systems biology, where molecular and cellular interactions can be studied across organs, and the relationship between structure and function can be explored at larger scales [6]. Additionally, spatially resolved transcriptomics, especially fluorescence in situ hybridization (FISH) and in situ sequencing (ISS) can give “barcodes” to cells, adding even more functional information to each image [7], [8]. Just as diseases affect the inner function of individual cells, diseases can also bring subtler changes in different but interacting cell populations over whole organs and organisms. Tissue clearing, fast 3D microscopy and more targeted fluorescent staining are now allowing us to study these large-scale systems [2].

However, new data analysis tools are needed to extract information from these large multi-channel 3D datasets. To quantify protein expression levels across organs, or to determine how diseases affect cell migration or cellular differentiation, tasks such as segmentation (e.g., cell, tissue, layer), tracking, and spatial analysis of fluorescent markers have to be performed. And with hundreds of thousands of cells per mm^3^ of tissue, such data processing efforts would be overly time-consuming and impractical if performed manually on large 3D image sets. Automatic data processing is thus essential to obtain quantitative measurements for analysis.

The spatial organization of cells plays an important role both in healthy and diseased organs, and studying cell organization would further our understanding of embryonic development, aging, disease pathways, and treatment strategies. However, few studies have looked into quantifying such organizations, with most experiments focusing on qualitative observations, or quantitative results obtained from a few cells. Fields such as graph theory and spatial statistics include many formal techniques to characterize spatial point distributions. Groups are starting to use such tools to analyze cellular distributions [9]–[13], but they require the location of each cell. The powerful toolsets of graph theory and spatial statistics could be used to study the 3D architecture of tissue at the cellular level if a robust 3D centroid detection algorithm could obtain a reliable spatial distribution.

In practice, 3D cell detection can be complicated by many factors. Whole tissue, organs and embryos have a multitude of different cell types, which are varied in shape and size, and have heterogeneous appearances when stained with fluorescent markers. Thus, few assumptions should be pre-built in cell detection algorithms. Additionally, low magnification microscope objectives have poor resolution in the depth axis, and the out-of-plane signal can make segmentation difficult. This is especially true since many existing algorithms were developed for 2D images of tissue sections and cell monolayers. However, using high magnification objectives with superior depth sectioning is not always an appropriate solution, since low magnification objectives have the long working-distances necessary for imaging deep in tissue, and their large field-of-view enables faster image acquisition. Additionally, imaging whole organs with high magnification objectives would generate a significant data burden.

An optimal 3D cell detection algorithm would accurately find the centroid position of cells in unsectioned tissue. It would perform optimally even on images acquired with low magnification objectives regardless of cell type, shape, and size. It would also translate easily from sample to sample within a study, and even adapt to different organs or animal models. Accurate detection of cell centroids would also enable spatial analysis of cell organization.

In this study we:

1. Propose a new segmentation-regression network to locate nuclei centroids in high density intact tissue.
2. Demonstrate the robustness of our approach by testing it in two different tissue types (cardiac embryonic tissue and brain stem tissue).
3. Perform spatial statistics analysis to determine which nuclei detection approach best preserves the true spatial organization of each tissue type.

## II. RELATED WORK

Multiple methods for automatic nuclei detection have been developed for different imaging modalities, including approaches designed for volumetric datasets. Before the recent advances in deep learning, approaches for cell segmentation included nuclei thresholding, matching predetermined templates to the image, the watershed transform, and deformable models [14]. To improve watershed results, algorithms have been developed to either identify seeds, or to iteratively refine the results based on the expected size and shape of the segmented objects [11], [15]. In the case of non-spherical nuclei found in muscle cells, complicated sequences of ellipse fitting, ellipse refinement and clustering have been used [16]. However, with the growing availability of fast and affordable graphics processing units (GPUs), approaches entirely based on deep-learning have become increasingly popular.

Deep learning is a promising approach when it comes to identifying objects with very few predetermined characteristics such as the nuclei of multiple cell types, which are of varied shapes and sizes. Most nuclei detection algorithms can be separated into two classes: either one attempts to segment each nucleus, or nucleus centroids can be directly detected without the need for prior object segmentation. The first option can provide many characteristics for each nucleus in addition to segmentation, such as the centroid location, the nucleus volume, and nucleus orientation. However, the second option may perform better in datasets where nuclei are clustered or overlapping, and where accurate segmentation is difficult. Recently, a third option has appeared, with multiple algorithms combining both a segmentation arm and a centroid detection arm, thus benefiting from the strengths of both approaches.

We now review recent studies falling within these three broad categories.

Many deep learning nuclei segmentation approaches in 2D and 3D are based on the U-net architecture [17]. U-net implementations are usually trained on two pixel classes, nucleus and background, and as a result are not separating touching nuclei without additional processing. In early publications, a thin line of “background” pixels was used to separate neighboring cells, and these pixels were heavily weighted during training to improve the final segmentation[17],[18].U-net implementations with three pixel classes (nucleus, nucleus boundary, background) have also been used in 2D with or without a variable weight distribution for training [19], [20]. The three pixel classes concept has also been extended to 3D, and has provided good segmentation results when paired with vectors pointing toward the nuclei center during training [21]. U-net has also been combined with other approaches, such as Mask R-CNN to separate clusters of nuclei in histology slides and 2D fluorescence images [22], and Hessian analysis to separate connected objects in 2D and 3D datasets [23].

Nuclei segmentation approaches have also used other neural network architectures beside U-net to segment nuclei. The idea of boundary detection was built in DCAN, a network with two output branches for segmenting nuclei and nuclei boundary separately in histology images [24]. Segmentation networks inspired by the 3D U-net architecture have also been proposed, such as DeepSynth which segments nuclei after training on synthetic images, and separates touching nuclei with a 3D watershed transform [25]. The V-net architecture [26], which is similar to 3D U-net but uses the Dice loss to prevent class imbalance, has also been used for cell segmentation using a multi-class approach to detect cells and cell boundaries [27].

Centroid locations can be calculated once nuclei are segmented, or they can be detected directly by a network. Regression neural networks have been used to find object positions, where they can be easier to train than classification networks [28], [29]. For cell detection, a centroid probability map can be calculated by a network, with the local maxima identified in post-processing indicating the cell centroid. A regression approach to detect nuclei has been advocated for by Kainz *et al.* who compared classification and regression approaches in random forests to localize cells in histology images [30]. Regression also performed well in convolutional neural networks [31], [32], fully convolutional networks (FCN)[33], and residual networks [34], especially on high cell density 2D images with clustered or overlapping objects. Regression-based networks were also robust and translated easily between different imaging modalities and cell types [33], [34]. Höfener *et al.* systemically tested different FCN implementations for centroid regression in histology images, and highlighted the importance of the post-processing steps to best identify centroids, recommending median filtering and Gaussian smoothing [35]. Finally, Hirsh and Kainmueller compared the centroids predicted by their proposed nuclei segmentation approach to a regression network inspired by Höfener *et al.* and found the regression method performed similarly to their own approach [21].

Recently, different groups have proposed pairing segmentation and centroid detection algorithms to improve accuracy, especially volumetric datasets with high nuclei density. One approach has used two 3D U-net networks, one to segment nuclei and the other to detect their centroid to improve segmentation accuracy in embryo images [36]. Another network, SphEsNet, was also inspired by a 3D U-net architecture and added small centroid detection and radius estimation branches to predict the size of spherical nuclei. This algorithm was also able to improve detection accuracy over methods such as Mask R-CNN and a previous network proposed by the authors [37]. Finally, a 3D U-net inspired segmentation network was combined with a small regression-based centroid detection branch and obtained an F1-score of 82% in a high nuclei density 3D fluorescent image set, and 93% in a low nuclei density data set [38].

Although regression-based approaches have proven to be especially strong at cell-detection tasks in 2D images, few attempts have been made in 3D datasets. Additionally, when segmentation and detection approaches have been paired, the detection branches were usually composed of a few convolutional layers at most, and not similar to the highly accurate regression networks used in 2D [37], [38]. In this manuscript we propose a new segmentation-regression approach that can be applied in intact cardiac and brain tissues, which both include irregularly shaped and clustered nuclei. We also compare our own segmentation-regression approach to other previously explored methodology, such as the inclusion of a nuclei-boundary pixel class to improve nuclei centroid detection.

## III. PROPOSED MODEL

### A. LEARNING TASK AND PROPOSED APPROACHES

Nuclei segmentation techniques have often been developed with the assumption that nuclei are spherical objects that do not significantly touch or overlap. In volumetric images of intact cardiac tissue, many nuclei appear to be touching because of the high tissue density and out-of-focus signal from cells at various depths. Additionally, nuclei of muscle cells tend to be elongated and elliptical in shape, while surrounding nuclei can take various shapes. As in other muscle cells [16], numerous small areas of high DAPI intensity are present within the nuclei, which complicates intensity-based thresholding and other segmentation algorithms that require homogeneous intensity. For these reasons, many existing cell segmentation and cell counting approaches lead to merged, split or missed nuclei, and inaccurate nuclei counts.

In this manuscript, five different approaches were tested to identify nuclei centroids in the DAPI stained embryonic heart wall:

1. VRegNet: V-net with two classes followed by a regression neural network,
2. Vnet-3: V-net with three classes and centroid calculation,
3. Vnet-3W: V-net with three classes followed by 3D watershed and centroid calculation,
4. Vnet-2W: V-net with two classes followed by 3D watershed and centroid calculation,
5. Segmentation and nuclei labeling in CellProfiler followed by centroid calculation.

**FIGURE 1.**
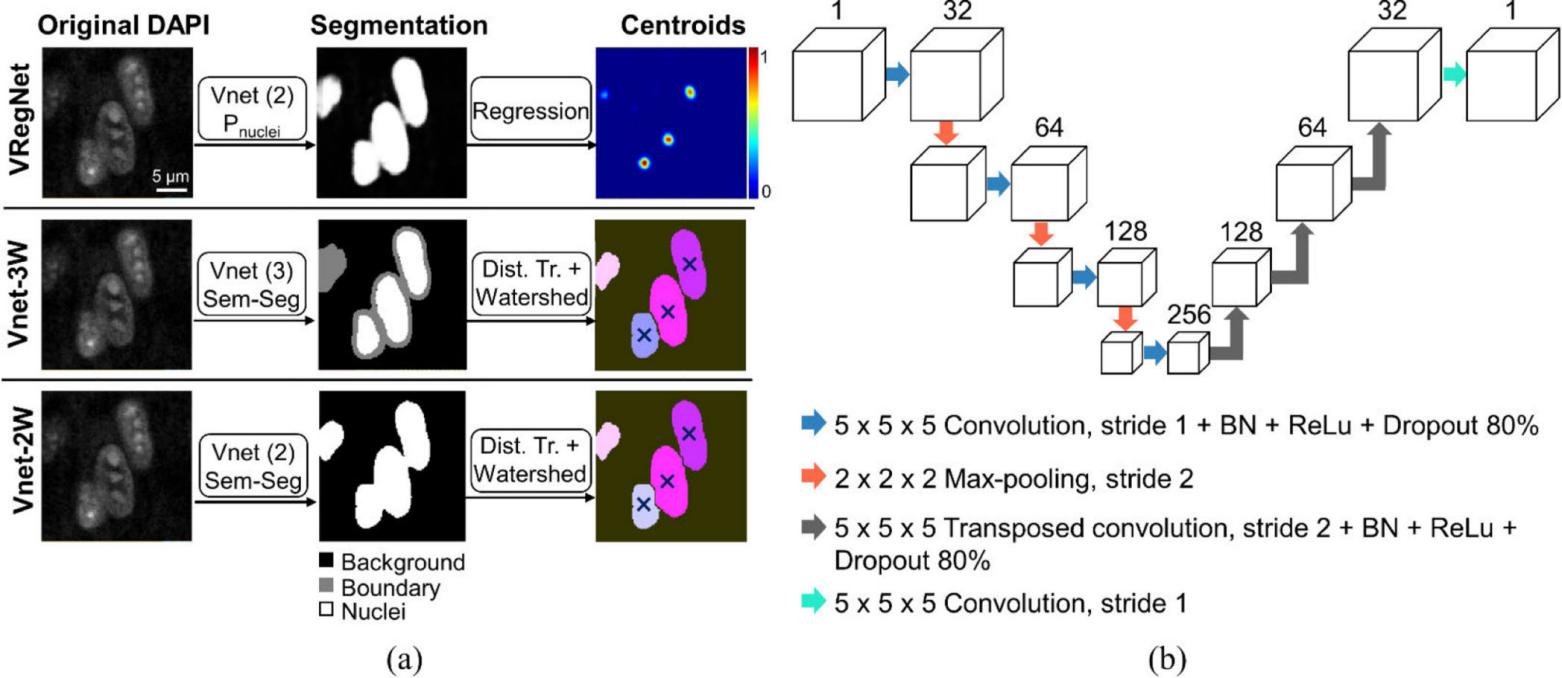
Visual summary of methods and the network architecture. (a) Two-step process to segment nuclei and identify centroid locations using a combination of a two-class V-net and a regression network (first row), a three-class V-net followed by a distance transform and 3D watershed (second row), or a two-class V-net followed by a distance transform and 3D watershed (third row). (b) Architecture of the regression network part of VRegNet. Vnet (2): two-class V-net. Vnet (3): three-class V-net. P_nuclei_: Probability of voxel being a nucleus. Sem-Seg: semantic segmentation. Dist. Tr.: distance transform. BN: batch normalization. ReLu: rectified linear unit.

VRegNet is our proposed two-step approach where nuclei are first segmented from the background using V-net, followed by a regression network to directly identify centroids. The other four methods are inspired by promising cell segmentation approaches found in the literature. Vnet-3 is a variation on the more traditional segmentation-based method, where in addition to the usual two classes used for segmentation (background vs nucleus), a third category (nucleus boundary) is introduced in an effort to separate touching nuclei. This approach has proven effective in 2D [20] and has been attempted in 3D [21], [27]. In Vnet-3W, Vnet-3 is combined to 3D watershed in post-processing to increase the separation between nuclei even more. To understand if the three-class approach provides any improvement over the more commonly used two-class approach (background vs nucleus), Vnet-2W functions as a stand-in for the many neural networks that have been proposed where nuclei are first segmented from the background, then separated using watershed [22], [25], [39]. A visual summary of VRegNet, Vnet-3W and Vnet-2W can be seen in Fig.1a. Finally, nuclei segmentation is also attempted using CellProfiler 4.0, a commonly used open-source software for cell segmentation, and a non-deep learning alternative that requires no upfront training.

### B. SEGMENTATION USING V-NET

V-net [26] is a fully convolutional network built for volumetric image segmentation, and is used as the main segmentation network in our first four approaches. It uses a Dice loss layer to prevent class imbalance, an important factor since the class “nucleus boundary” has the fewest voxels while having an essential role in separating overlapping nuclei. We found during training that V-net performed similarly or slightly better at segmenting cardiac nuclei than other common 3D segmentation networks such as 3D U-net. We thus decided to use V-net for all approaches to make comparisons easier. We used a MATLAB (MathWorks, Natick, MA, USA) implementation of V-net [40].

### C. REGRESSION NETWORK

The regression network in VRegNet (Fig.1b) is a simple encoder-decoder network, inspired by Xie et al [33] with 2D convolution layers extended to 3D, and with added batch-normalization and dropout layers to regularize training and prevent overfitting. The convolution layers have a size of 5 × 5 × 5 voxels with a stride of 1 and zero-padding of 2 voxels around all edges. The transposed convolution layers also have a size of 5 × 5 × 5 voxels with a stride of 2. The result of each layer is cropped so that in each dimension the output is double the input in size. Weights were initialized using the Glorot initializer [41], and biases were initialized to zero.

The central task of the regression network is to determine the centroids’ position from the nuclei image. However, contrary to examples from previous work where the regression network was applied directly to the DAPI image [33], the input of our network is the output of the two-class V-net before the final voxel classification layer (i.e., the probability of each voxel belonging to a nucleus). This first step homogenizes the appearance of the nuclei and simplifies the regression network’s task.

To train the regression network, a centroid probability map is created by placing a small 3D Gaussian (e.g., standard deviation: 3 voxels) at the location of each centroid in the input volume to serve as the ground truth. During training, the loss is calculated as the mean square error between the ground-truth probability map and the predicted probability map. In post-processing, the local maxima of the probability map are identified as the centroid locations.

### D. CELLPROFILER

Nuclei segmentation was performed in CellProfiler 4.0, a widely used open-source software. “Optimal” settings were found through trial-and-error and visual assessment on each testing dataset. First, a morphological opening operation was performed on the DAPI image with a disk structuring element. This homogenized the appearance of each nucleus. Then Otsu thresholding was performed to separate the background from the nuclei. A 3D watershed transform was performed on the distance map of the binary image, and each cell was then labeled with a unique identifier. Centroid position for each of the labelled nuclei was then calculated in MATLAB.

## IV. EXPERIMENTAL METHODS

### A. SAMPLE PREPARATION AND DATA ACQUISITION

Quail hearts at embryonic development day 9 were surgically removed, fixed overnight in 4% paraformaldehyde at 4°C, then stained for 72h with DAPI (1mg/mL in 1% PBS). Optical clearing was performed at room temperature using LIMPID [42]. Three-dimensional image stacks of the posterior left ventricle wall were acquired on an inverted confocal microscope (SP8 with HyVolution 2, Leica Microsystems Inc., Buffalo Grove, IL, USA) using a 20x/0.75 NA objective set for glycerol immersion. Voxel size was 177.6 × 177.6 nm × ∼1.06 *μ*m (x, y, z) and optical sectioning was 3.12*μ*m.DAPIimageswereacquiredusing405nmexcitation and 416–490 nm emission. The excitation light intensity was progressively increased with depth to compensate for tissue absorption.

Brain stem from adult mice (C57BL/6) were surgically removed, fixed for two hours in 4% paraformaldehyde at room temperature, then vibratome sliced, stained with DAPI (10 ng/mL in 1% PBST), and cleared using LIMPID. Three-dimensional image stacks were acquired on the same microscope and objective as the quail heart sample, with a voxel size of 0.75 × 0.75 × 0.8 *μ*m (x, y, z).

All procedures were performed in accordance with relevant guidelines and regulations under the approval of the Case Western Reserve University Institutional Animal Care and Use Committee (IACUC). IACUC approval was not required for the quail embryos as the Policy for use of Avian embryos at Case Western Reserve University states “if embryos will be sacrificed prior to 3 days before hatching, the research will not be subject to IACUC review.”

### B. GROUND TRUTH CREATION

The training dataset (400 × 200 × 129 voxels, approximately 200 nuclei) and validation dataset (200 × 200 × 129 voxels, ∼100 nuclei) were chosen from one embryonic quail heart and each nucleus was manually segmented and assigned a unique label in ITK-SNAP [43]. Three ground truth datasets were created: 1) a two-label (background/nuclei) volume, 2) a three-label (background/boundary/nuclei) volume where a three-voxel wide boundary surrounded each nucleus, and 3) a nucleiprobabilitymapwith3DGaussians(standard deviation 0.5 *μ*m) located at each nuclei centroid.

Testing datasets were created from the same embryonic quail heart (heart #1, 700 × 700 × 150 voxels), a second quail heart of the same developmental stage but imaged in a separate session (heart #2, 390 × 390 × 150 voxels), and a mouse brain stem sample (brain #1, 390 × 390 × 265 voxels). Small training and validation sets from brain #1 (200 × 390 × 36 voxels each) were also manually segmented and annotated for fine-tuning of the brain stem data. Nuclei centroids were manually identified by one user using ITK-SNAP in all datasets and were considered as the ground truth. Two more users also annotated all centroids in heart #1 to estimate inter-user variability. It was determined that different users positioned centroids on average 0.54 ± 0.36 *μ*m away from other users, with no significant bias in any directions.

### C. DATA AUGMENTATION AND PATCH SELECTION

Small volume-patches were extracted randomly from the larger training volume every iteration. In order to expand the size of the training dataset and prevent overfitting, data augmentation was performed on-the-fly on each patch during training. One of following four operations was randomly assigned: reflection with respect to the x or y axis, volume rotation by 90° (counterclockwise) around the z-axis, or a combination of reflection followed by 90° rotation. One of these four operations was applied 50% of the time, and no augmentation was applied the other 50% of the time.

### D. DEEP LEARNING EXPERIMENTS

All experiments were implemented in MATLAB 2020a. The computer had 256 GB of memory, a 24 core CPU at 2.2 GHz, and an 11 GB GeForce RTX 2080Ti GPU (NVIDIA Corporation, Santa Clara, CA, USA).

Three networks in total were trained on the same training and validation dataset: 1) the regression network part of VRegNet, 2) the two-class V-net part of VRegNet and Vnet-2W, and 3) the three-class V-net part of Vnet-3 and Vnet-3W. The training parameter space was explored and the best parameters for each network were chosen based on the segmentation accuracy and centroid placement achieved on the validation set. The best network parameters found are listed in Table 1. The Adam optimizer [44] was used with default parameters. Training was stopped if the validation loss did not decrease for more than two epochs, or increased for more than one epoch. Since a random image patch was extracted from the training volume every iteration, the total iteration number is equal to the number of unique image volumes seen by the network during training.

The regression network can be difficult to train when most voxels in the ground-truth probability map are equal to zero, with few centroids corresponding to local peaks equal to 1 in intensity (see Fig.1a, VRegNet/centroids panel). To facilitate network convergence and avoid predicting every voxel to the background value of zero, the ground-truth probability map was multiplied by a factor of 10,000 during training, which forces the network to fit the Gaussians [33].

### E. DATA POST-PROCESSING

In VRegNet, the nuclei were identified in the original DAPI volume using a two-class V-net one patch at a time. The whole output volume of the V-net was median filtered (filter size: 3 × 3 × 3 voxels) to smooth the transition between neighboring patches while preserving the nuclei feature, then passed onto the regression network. Once the output of the regression network was obtained, the local maxima in the centroid probability map were detected. First, an intensity threshold was applied to the probability map to set background values to zero. The volume was then filtered using a 3D gaussian kernel (standard deviation: 4 × 4 × 2 voxels), taking care to smooth each of the peaks without merging them with their neighbors. Local maxima within a 2 *μ*m radius region were then identified as centroids and their locations recorded.

In Vnet-3W and Vnet-2W, the nuclei in the original DAPI volume were identified using the two-class or three-class V-net network. The voxels identified with a probability *>* 0.5 of belonging to a nucleus were separated from the background. The segmented volumes were smoothed using a 3D gaussian filter (standard deviation: 5 × 5 × 5 voxels) and resampled so that voxels were isometric (tricubic interpolation). A distance map was then calculated on each binary volume, and a 3D watershed transform was applied to label all nuclei. The centroid position was then calculated for each labeled nucleus.

Each of the five approaches, but especially VRegNet, did not perform optimally when nuclei were intercepted by the edges of the volume, since it is difficult to identify the centroid of an incomplete object. To solve this issue, a 30 × 30 × 5 voxel border was removed from each volume in post-processing, and nuclei in this region were not considered during analysis.

### F. SUCCESS METRICS FOR CENTROID DETECTION

Predicted centroid locations were compared to the ground-truth to quantify the accuracy of each approach. Cardiac nuclei were approximately 2–4 *μ*m in radius, while brain stem nuclei were approximately 3–6 *μ*m in radius depending on the cell type and cell orientation. A centroid predicted by one of the five approaches was thus considered as a true positive (TP) if its position was within 3 *μ*m of the ground-truth centroid. Ground-truth centroids with no corresponding predicted centroid were considered false negative (FN). Predicted centroids that were positioned further than 3 *μ*m from any ground-truth centroids were considered false positive (FP). Additionally, if two or more centroids were predicted within the 3 *μ*m boundary of the same ground-truth centroid, the closest would be considered as TP, and the others as FP.

The precision (P), recall (R), and F1-score were calculated as follow:

(1) $$P=\frac{TP}{TP+FP}$$

(2) $$R=\frac{TP}{TP+FN}$$

(3) $$F1=\frac{2PR}{P+R}$$

When a centroid was determined to be a TP, the distance between the predicted and ground-truth centroid was calculated. The predicted cell count was obtained by adding the number of TP centroids to the FP number. The cell count error was calculated as the true cell count subtracted from the predicted cell count, divided by the true cell count.

## V. RESULTS AND ANALYSIS

### A. COMPARISON OF CENTROID-FINDING NETWORKS IN CARDIAC TISSUE

**FIGURE 2.**
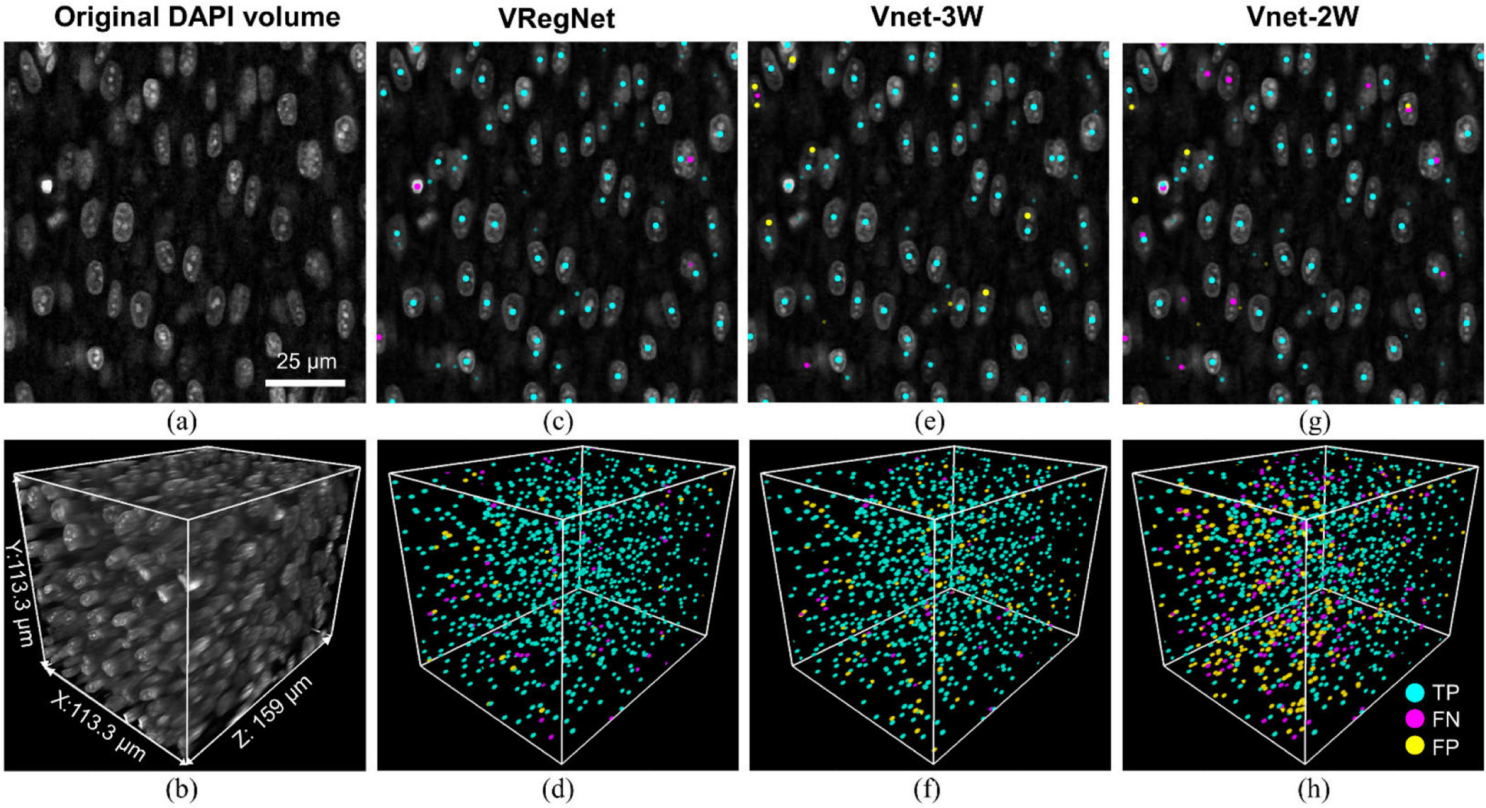
Nuclei centroid identification in a quail heart at embryonic day 9. (a) 2D slice from the original DAPI volume. (b) 3D rendering. (c-d) 2D and 3D display of true positive (TP), false negative (FN) and false positive (FP) centroids obtained with VRegNet, (e-f) Vnet-3W, (g-h) Vnet-2W.

The performance of the five different approaches was first evaluated in the intact, optically cleared, DAPI stained quail embryonic heart (heart #1). The testing volume was from the same heart as the volume used for training, and both were collected in the posterior left ventricular wall. A representative 2D slice and a 3D rendering of the test volume can be seen in Fig.2a, b. The test volume was acquired starting at approximately 5 *μ*m in depth from the epicardium, to approximately 164 *μ*m in depth. The location of the TP, FP and FN centroids predicted by VRegNet, Vnet-3W, Vnet-2W are marked for the same 2D slice and 3D volume in Fig.2c-h (see also supplementary videos 1-3). The 2D slice shown (Fig.2a, c, e, g) was acquired at 115 *μ*m in depth. It can be seen that while VRegNet tends to miss a few nuclei (FN, magenta, Fig. 2c), Vnet-3W more often double-counts nuclei (FP, yellow, Fig. 2e) and Vnet-2W both misses some nuclei (FN, Fig. 2g) and falsely labels background voxels as centroids (FP, Fig. 2g).

The quantitative analysis of each approach’s performance can be seen in Table 2 for heart #1. VRegNet had the highest F1-score and the most accurate cell count. However, Vnet-3W had the smallest distance between ground-truth and predicted centroids. Vnet-2W had a low F1-score compared to VRegNet and Vnet-3W. Both Vnet-3W and Vnet-2W over-counted the centroids, often by assigning more than one centroid per nucleus. On the other hand, Vnet-3 (no watershed) and the CellProfiler approaches were both very likely to merge nuclei and thus undercount them.

The performance of all five approaches was also evaluated on a second embryonic heart (heart #2), which was imaged on the same microscope with similar image settings, but on a different day as heart #1. This second sample tested how well the different neural networks performed on a new sample on which they had not been trained. The success metrics for heart #2 can be seen in Table 3. The F-1 score for VRegNet has decreased slightly, while it has increased slightly for Vnet-3W. The cell count is also closer to the ground truth in Vnet-3W than in VRegNet. The precision of Vnet-2W has increased; however, the network is now undercounting the nuclei.

The results from Table 3 were obtained without fine-tuning or transfer learning to adapt to the new dataset. This indicates generalization and robustness of both VRegNet and Vnet-3W. Optimally, one could analyze multiple samples of the same cohort for a larger study without requiring any additional training from one sample to another. Fine-tuning of the regression and V-net networks was attempted using a small 200 × 390 × 32 voxels training sub-volume from heart #2, but this did not lead to any noticeable improvement amongst the centroid detection metrics. We thus report the result without fine-tuning.

### B. COMPARISON OF CENTROID-FINDING NETWORKS IN BRAIN STEM TISSUE

**FIGURE 3.**
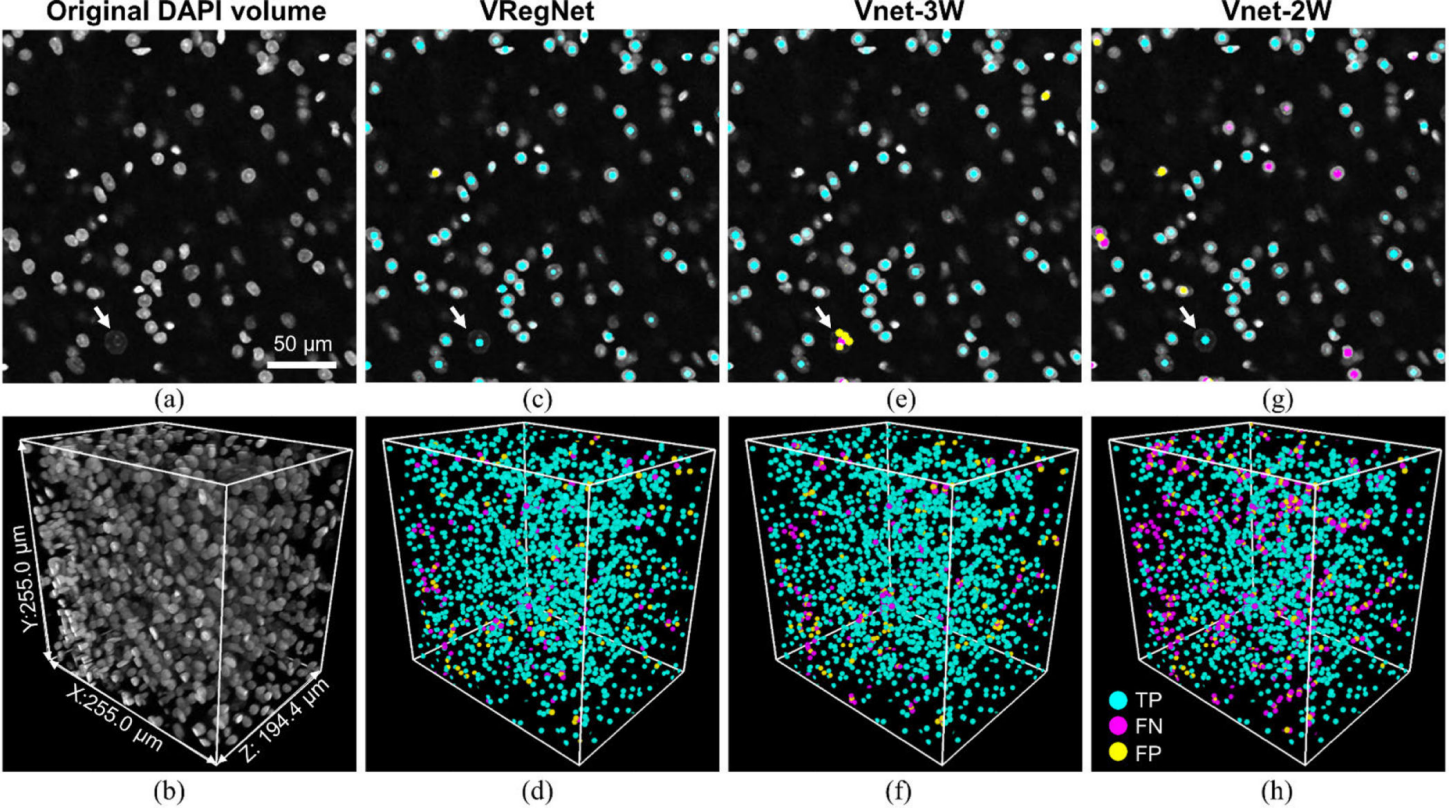
Nuclei centroid identification in an adult mouse brain stem. (a) 2D slice from original DAPI volume. (b) 3D rendering. (c-d) 2D and 3D display of true positive (TP), false negative (FN) and false positive (FP) centroids obtained with VRegNet, (e-f) Vnet-3W, (g-h) Vnet-2W. White arrow: example neuron nucleus which is larger and low in intensity compared to other nuclei.

Ideally, networks trained for centroid detection in one tissue type would be robust and easily adaptable to different samples and tissue types without the need for significant retraining, which would require a large time commitment for manual ground-truth labeling. We thus tested each of the five approaches on a different tissue type: the adult mouse brain stem. The brain stem sample was optically cleared and DAPI stained similarly to the quail embryonic hearts; however, it was imaged with a different voxel size, on a different day, and by a different user who manually adjusted parameters such as excitation intensity. The brain stem cell nuclei are also more spherical and homogeneous in appearance. Additionally, the sample includes neuron nuclei, which are significantly larger with lower fluorescent intensity than the surrounding cells (see Fig. 3a, arrow). Many nuclei are also clumped, which can make them difficult to segment with traditional techniques. A 2D slice of the brain stem data and a 3D rendering of the test volume can be seen in Fig 3a, b.

As mentioned in section IV. B, small training and validation sets from the brain stem data were used for fine-tuning the regression and V-net networks. Without this fine-tuning, the F1-scores for VRegNet, Vnet-3W and Vnet-2W were 65.57%, 87.97% and 80.35% respectively. The test volume was kept separate from the training and validation sub-volumes used for fine-tuning. To facilitate transfer learning, each patch from the brain stem data was resized during training in the x-y dimensions to three times its original size, to better match the apparent size of the cardiac nuclei. For the regression network, a learning rate of 5e-5, with a learning rate drop factor of 0.8 and a period of 5 epochs was chosen. It trained for 45 epochs. For the three-class V-net and two-class V-net, the learning rate for fine-tuning was 5e-5, with a drop factor of 0.8 and a period of 5 epochs. They trained for 10 epochs after which no further improvement in accuracy could be seen. The location of the TP, FP and FN centroids predicted by VRegNet, Vnet-3W and Vnet-2W after all networks were fine-tuned are marked for a 2D slice and 3D volume in Fig.3c-h.

VRegNet performed well with few FP and FN seen in Fig. 3c-d. Vnet-3W also performed well, but had particular difficulties identifying neuron nuclei (Fig. 3e, white arrow). Vnet-2W merged many close nuclei, which lead to many two FNs (magenta) surrounding one FP (yellow), as seen in Fig. 3g-h.

The quantitative evaluation of the different approaches in the brain stem volume can be seen in Table 4. For each approach, the F1-scores and cell count errors are similar to the ones obtained in heart #1, with VRegNet and Vnet-3W performing best. The distance between predicted and ground-truth centroids were also similar between approaches. Surprisingly, CellProfiler performed better on this sample than on both cardiac samples, possibly because the spherical shape of the nuclei was similar to the type of samples for which CellProfiler is optimized. However, it still undercounts nuclei by 20% and has the worst centroid distance of all methods.

Overall, both VRegNet and Vnet-3W consistently had high F1-scores, low centroid distance and accurate cell counts on all embryonic quail heart and adult mouse brain stem datasets, followed by much lower scores using Vnet-2W. These results were achieved with no fine tuning after the original network training in the heart data, or minimal fine tuning on a small volume when adapting the networks to a different tissue type. By being robust and flexible, both VRegNet and Vnet-3W have strong potential for routine image analysis in a biological research setting.

### C. PROCESSING TIMES

Times required to detect nuclei in heart #1 (700 × 700 × 150 voxels, 993 nuclei) were compared for all methods. The results can be seen in Table 5, and are broken down in three sub-categories. Nuclei segmentation was accomplished using the two-class V-net (VRegNet and Vnet-2W) or the three-class V-net (Vnet-3W and Vnet-3). Instance detection was performed using the regression neural network in VRegNet to detect centroids, and the watershed transform in Vnet-3W and Vnet-2W to label individual nucleus. In Vnet-3, connected components were used to label individual nucleus. CellProfiler performed both nuclei segmentation and the watershed transform in one process. Finally, centroid detection was performed either by identifying local maxima for VRegNet, or calculating the centroid of each labeled nuclei for all other methods. All times shown are the mean of five trials.

As seen in Table 5, VRegNet is significantly faster than other methods by directly detecting the centroids of nuclei instead of relying on the watershed transform. It can accurately detect nearly a thousand nuclei in under a minute. However, VRegNet was mostly performed on a GPU, which accelerated the process compared to Vnet-3W, Vnet-2W and CellProfiler, which executed the watershed transform on a CPU.

### D. SPATIAL STATISTICS OF 3D NUCLEI DISTRIBUTION

In this manuscript, we present different approaches that would replace manual cell identification and permit spatial statistical analysis in organs such as the developing heart and the brain stem. For those automated approaches to be most useful, they would need to replicate as closely as possible the spatial distribution of manually identified cells. We thus compared all five approaches to the manually segmented ground truth using three common spatial statistical functions: the F-function, the G-function and the H-function [45]. Each function was calculated in 3D using the Spatial Statistics 2D/3D plugin in ImageJ [9], [46], [47].

**FIGURE 4.**
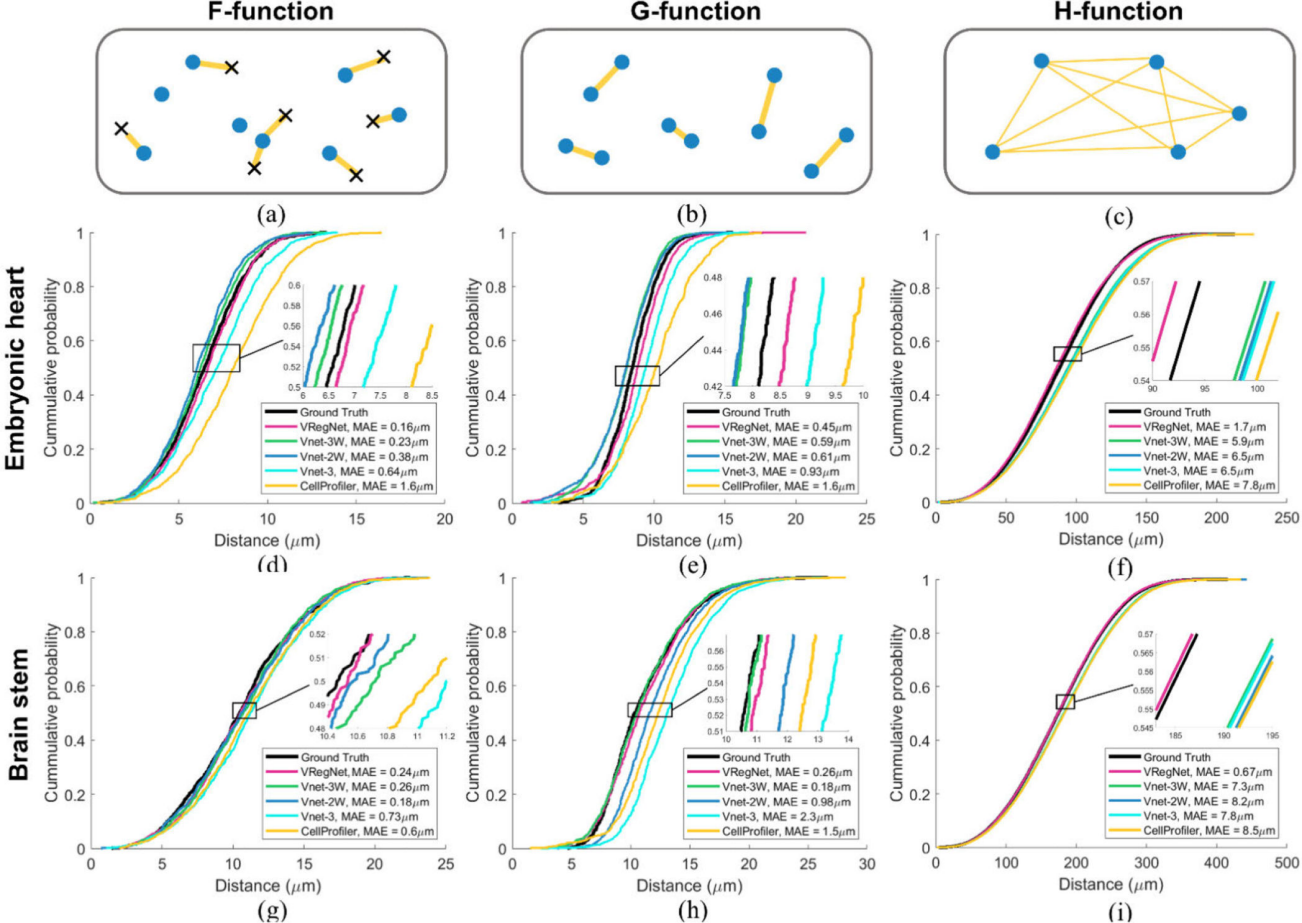
Spatial statistics analysis of nuclei centroid detection approaches. Graphical representation of the centroids (blue), with random locations (x) selected, and the distribution of distances (yellow) represented for the (a) F-function, (b), G-function, and (c) H-function. (d) F-function, (e), G-function, and (f) H-function calculated for each centroid-detection approach in the embryonic quail heart (heart #1), and in a mouse brain stem (brain #1) (g-i). MAE: mean absolute error, calculated between each approach and the ground truth curve.

The F-function describes the cumulative probability for a randomly chosen location anywhere in the test volume to be within a certain distance of the nearest centroid as represented in Fig.4a. The G-function describes the cumulative probability of the average centroid to be within a certain distance of its nearest centroid neighbor as seen in Fig.4b. Finally, the H-function, which describes the cumulative probability of a centroid to be at a certain distance from any other centroids in the volume as can be seen in Fig.4c.

The F, G and H-functions for the ground-truth and all five approaches for the embryonic cardiac nuclei (heart #1) can be seen in Fig.4d-f, including the mean absolute error (MAE) between each approach and the ground truth. For each spatial statistics function, VRegNet most resembles the probability distribution of the ground-truth followed by Vnet-3W. In the F-function, curves are shifted to the right when a method undercounts cells, since it leads to more empty space in the image volume, and thus a larger distance on average between a random location and the nearest centroid in the volume. A decrease in the curve’s slope would also indicate that regions without centroids have appeared in the volume more often than in the ground-truth because of errors in centroid identifications. In the G-function, curves are also shifted to the right when undercounting, since the average distance between nearest centroids is increased. A shift to the right could also indicate that centroids are more regularly spaced from each other, while a shift to the left could indicate clusters. In the H-function, both under- and overcounting leads to a shift to the right, since they are both more likely to increase the average distance between one centroid and all other centroids, than to decrease it.

The F, G and H-functions for the mouse brain stem data can be seen in Fig.4g-i, respectively. It can be seen from Fig. 3g, h that Vnet-2W tends to merge clustered nuclei, which affected the G-function by shifting it to the right without affecting the F-function. Vnet-3W also tends to merge clustered nuclei, but this effect is balanced by over-splitting larger neuron nuclei (Fig. 3e, white arrow), which creates incorrect centroid clusters. This may explain why Vnet-3W seems to perform well in the F and G-functions but fails to replicate the ground-truth distribution in the H-function.

When comparing the G-functions of the ground truth, we observed that 50% of nuclei were within 8.48 *μ*m of their nearest neighbor in the heart, while 50%ofnuclei were within 10.34 *μ*m of their nearest neighbor in the brain. This indicates that the cells in the embryonic quail heart might be more tightly packed than in the mouse brain stem. Those distances are 8.83 *μ*m and 10.74 *μ*m, respectively, when measured using the VRegNet G-functions, indicating that the difference between tissues is preserved.

In the cardiac embryonic dataset (heart #1), VRegNet most closely resembles the spatial pattern of the manually labeled ground truth in large part because it obtained the most accurate cell count, but also because it did so while maintaining the distribution of distances between centroids and their neighbors. VRegNet also performs well across all three functions in brain #1, even though it did not achieve the most accurate cell count. This demonstrates that the position of nuclei with respect to their neighbors, not just the total nuclei count, plays an important role when describing spatial distributions.

## VI. CONCLUSION

In this manuscript, we present VRegNet, a new combination of nuclei-segmentation and centroid-regression networks to improve detection of nuclei in large 3D fluorescence datasets. This new approach was able to detect centroids with high accuracy in both intact quail embryonic hearts and the mouse brain stem, even though the tissue types included clustered nuclei of different shapes, sizes and fluorescent intensity. VRegNet was robust and maintained similar accuracy when tested on a heart sample that was not included in the training dataset, and it easily adapted to the brain stem images with minimal fine-tuning. VRegNet also maintained the spatial distribution of the nuclei found in the manually segmented ground-truth data, and thus could be used in further studies of spatial cell patterns.

This is an additional demonstration that regression-based approaches provide high accuracy nuclei detection, including in 3D datasets. Nuclei centroids in 3D tissue images cannot be accurately positioned by a 2D centroid detection algorithm working on one image slice at a time. It is thus important that algorithms that have proven effective in 2D [30]–[34] are fully adapted to 3D image processing.

A V-net approach with three different classes (nucleus, nucleus boundary and background) followed by a 3D watershed transform was also highly effective at separating nuclei and obtained a higher accuracy when calculating the nuclei position in 3D. If desired for analysis, the three-class V-net would also provide more information about each nucleus (e.g., size, shape and orientation especially for the ellipsoid-shaped cardiac nuclei). However, we estimated it took approximately 5 times longer for an expert user to manually segment nuclei for three-class V-net training compared to manually indicating centroid position for the same dataset. It also took nearly 18 times longer to identify centroids in testing data (heart #1) with Vnet-3W than with VRegNet (see Table 5). A regression approach would thus be less time-consuming at the initial training stage, when adapting the networks from one tissue type to another, and when detecting nuclei with a trained network.

VRegNet successfully detected nuclei in two hearts imaged on different days and with slightly different imaging parameters (such as excitation laser power which was manually adjusted every imaging session). However, the network might need fine-tuning if samples were acquired on multiple microscopes, with different staining and clearing protocol, or with different objectives. Nonetheless, as shown in the brain stem sample that was acquired with different imaging parameters and voxel size, the architecture of VRegNet is robust and high-quality results can be obtained after network fine-tuning to new samples.

As indicated in Höfener *et al.*, it is likely that centroid detection would be improved by increasing the size of the training datasets [35]. Additionally, different post-processing steps to identify the local maxima of the centroid probability map could also improve our results without changing the regression network itself. We will explore those possibilities in future studies.

In conclusion, we present VRegNet, a novel combination of the segmentation architecture V-net, and our own 3D fully convolutional regression network as a robust and accurate method to locate nuclei centroids. Our method performed similarly or better to segmentation-based methods combined to 3D watershed, a common post-processing step. Additionally, VRegNet most closely resembled the spatial centroid distribution obtained from manual nuclei identification making it appropriate for future studies of tissue organization.

## Supplementary Material

access-3073894-mm

## Figures and Tables

**TABLE 1.** Training parameters.

Parameters	Regression	V-net (2)	V-net (3)
Initial learning rate	le-3	le-3	le-3
Drop Factor Drop Period (Epoch)	0.755	0.95	0.975
Mini-batch size	2	2	1
Patch size (x, y, z)	(200,200, 32)	(128,128, 32)	(128, 128, 32)
Patch/epoch	50	50	100
Total epoch #	15	51	10
Total iteration #	750	2550	1000
Optimization algorithm	Adam	Adam	Adam

Training parameters for the centroid regression network, the two-class V-net (nucleus/background), identified V-net (2) and the three-class V-net (nucleus/nucleus boundary/background), identified V-net (3). Patch size is given in units of voxels.

**TABLE 2.** Success Metrics: Heart #1.

Methods	TP	FP	FN	Precision	Recall	FI-score	Centroid distance (pm)	Cell count (true = 993)	Cell count error
VRegNet	940	39	53	96.02%	94.66%	95.33%	0.96 ±0.60	979	−1.41%
Vnet-3W	956	115	37	89.26%	96.27%	92.64%	0.72 ±0.55	1071	+7.85%
Vnet-2W	818	293	175	73.63%	82.38%	77.76%	0.93 ± 0.82	1111	+11.88%
Vnet-3	600	146	393	80.43%	60.42%	69.01%	0.91 ± 0.85	746	−24.87%
CellProfiler	219	279	774	43.98%	22.05%	29.38%	1.98 ± 1.01	498	−49.85%

Detected nuclei centroids in an embryonic quail heart for all approaches. TP: True positive. FP: False positive. FN: False negative. Distance between true centroids and detected centroids presented as mean centroid distance ± standard deviation.

**TABLE 3.** Success Metrics: Heart #2.

Methods	TP	FP	FN	Precision	Recall	FI-score	Centroid distance (μm)	Cell count (true = 412)	Cell count error
VRegNet	364	11	48	97.07%	88.35%	92.50%	1.02 ±0.63	375	−8.98%
Vnet-3W	398	13	14	96.84%	96.60%	96.72%	0.67 ± 0.50	411	−0.24%
Vnet-2W	336	15	76	95.73%	81.55%	88.07%	0.82 ± 0.76	351	−14.80%
Vnet-3	166	46	246	78.30%	40.29%	53.21%	1.01 ±0.96	212	−48.54%
CellProfiler	81	73	331	52.60%	19.66%	28.62%	2.18 ±1.01	154	−62.62%

Detected nuclei centroids for a second embryonic quail heart, which was not part of the training or validation set. TP: True positive. FP: False positive. FN: False negative. Distance between true centroids and detected centroids presented as mean centroid distance ± standard deviation.

**TABLE 4.** Success Metrics: Brain #1.

Methods	TP	FP	FN	Precision	Recall	FI-score	Centroid distance (nm)	Cell count (true = 1728)	Cell count error
VRegNet	1616	85	112	95.00%	93.52%	94.25%	0.94 ±0.59	1701	−1.56%
Vnet-3W	1583	127	145	92.57%	91.61%	92.02%	0.85 ± 0.50	1710	−1.04%
Vnet-2W	1362	162	366	89.37%	78.82%	83.76%	0.82 ±0.53	1524	−11.80%
Vnet-3	1044	237	684	81.50%	60.42%	69.39%	0.83 ± 0.54	1281	−25.87%
CellProfiler	1069	312	659	77.41%	61.86%	68.77%	1.03 ±0.57	1381	−20.08%

Detected nuclei centroids in a mouse brain stem sample after network fine-tuning. TP: True positive. FP: False positive. FN: False negative. Distance between true centroids and detected centroids presented as mean centroid distance ± standard deviation.

**TABLE 5.** Processing times.

Methods	Nuclesegmentation	Instance detection	Centroid detection	Total time
VRegNet	24.8 s	17.4 s	15.6 s*	57.8 s
Vnet-3W	24.8 s	11.0 min*	5.3 min*	16.7 min
Vnet-2W	24.7 s	12.1 min*	5.4 min*	17.9 min
Vnet-3	24.8 s	8.8 s*	3.8 min*	4.4 min
CellProfiler	11.1 min*		2.5 min*	13.6 min

Average times for each processing step in heart #1 (whole volume) over five timed trials for each method.

* Indicates processing performed on CPU instead of GPU.

## References

[1] Ueda HR, Ertürk A, Chung K, Gradinaru V, Chédotal A, Tomancak P, and Keller PJ, “Tissue clearing and its applications in neuroscience,” Nature Rev. Neurosci, vol. 21, no. 2, pp. 61–79, Feb. 2020, doi: 10.1038/s41583-019-0250-1. 31896771 PMC8121164

[2] Hillman EMC, Voleti V, Li W, and Yu H, “Light-sheet microscopy in neuroscience,”Annu.Rev.Neurosci,vol.42,no.1,pp. 295–313,Jul.2019, doi: 10.1146/annurev-neuro-070918-050357. 31283896 PMC6800245

[3] Wan Y, McDole K, and Keller PJ, “Light-sheet microscopy and its potential for understanding developmental processes,” Annu. Rev. Cell Develop. Biol, vol. 35, no. 1, pp. 655–681, Oct. 2019, doi: 10.1146/annurev-cellbio-100818-125311. 31299171

[4] Kolesová H, Čapek M, Radochová B, Janáček J, and Sedmera D, “Comparison of different tissue clearing methods and 3D imaging techniques for visualization of GFP-expressing mouse embryos and embryonic hearts,” Histochem. Cell Biol, vol. 146, no. 2, pp. 141–152, Aug. 2016, doi: 10.1007/s00418-016-1441-8. 27145961

[5] Cai R. , “Panoptic imaging of transparent mice reveals whole-body neuronal projections and skull–meninges connections,” Nature Neurosci., vol. 22, no. 2, pp. 317–327, Feb. 2019, doi: 10.1038/s41593-018-0301-3. 30598527 PMC6494982

[6] Susaki EA and Ueda HR, “Whole-body and whole-organ clearing and imaging techniques with single-cell resolution: Toward organism-level systems biology in mammals,” Cell Chem. Biol, vol. 23, no. 1, pp. 137–157, Jan. 2016, doi: 10.1016/j.chembiol.2015.11.009. 26933741

[7] Maniatis S, Petrescu J, and Phatnani H, “Spatially resolved transcriptomics and its applications in cancer,” Current Opinion Genet. Develop, vol. 66, pp. 70–77, Feb. 2021, doi: 10.1016/j.gde.2020.12.002. PMC7969406 33434721

[8] Asp M, Giacomello S, Larsson L, Wu C, Fürth D, Qian X, Wärdell E, Custodio J, Reimegård J, Salmén F, Österholm C, Ståhl PL, Sundström E, Åkesson E, Bergmann O, Bienko M, Månsson-Broberg A, Nilsson M, Sylvén C, and Lundeberg J, “A spatiotemporal organwide gene expression and cell atlas of the developing human heart,” Cell, vol. 179, no. 7, pp. 1647.e19–1660.e19, Dec. 2019, doi: 10.1016/j.cell.2019.11.025. 31835037

[9] Andrey P, Kiêu K, Kress C, Lehmann G, Tirichine L, Liu Z, Biot E, Adenot P-G, Hue-Beauvais C, Houba-Hérin N, Duranthon V, Devinoy E, Beaujean N, Gaudin V, Maurin Y, and Debey P, “Statistical analysis of 3D images detects regular spatial distributions of centromeres and chromocenters in animal and plant nuclei,” PLoS Comput. Biol, vol. 6, no. 7, Jul. 2010, Art. no. e1000853, doi: 10.1371/journal.pcbi.1000853. PMC2900307 20628576

[10] Diggle PJ, “Displaced amacrine cells in the retina of a rabbit: Analysis of a bivariate spatial point pattern,” J. Neurosci. Methods, vol. 18, nos. 1–2, pp. 115–125, Oct. 1986. 3796038 10.1016/0165-0270(86)90115-9

[11] Nhu HTT, Drigo RAE, Berggren P-O, and Boudier T, “A novel toolbox to investigate tissue spatial organization applied to the study of the islets of langerhans,” Sci. Rep, vol. 7, no. 1, pp. 1–12, Mar. 2017, doi: 10.1038/srep44261. 28303903 PMC5355872

[12] Lund AW, Bilgin CC, Hasan MA, McKeen LM, Stegemann JP, Yener B, Zaki MJ, and Plopper GE, “Quantification of spatial parameters in 3D cellular constructs using graph theory,” J. Biomed. Biotechnol, vol. 2009, Nov. 2009, Art. no. 928286, doi: 10.1155/2009/928286. PMC2775910 19920859

[13] Acar E, Plopper GE, and Yener B, “Coupled analysis of in vitro and histology tissue samples to quantify structure-function relationship,” PLoS ONE, vol. 7, no. 3, Mar. 2012, Art. no. e32227, doi: 10.1371/journal.pone.0032227. PMC3316529 22479315

[14] Meijering E, Dzyubachyk O, Smal I, and van Cappellen WA,”Tracking in cell and developmental biology,” Seminars Cell Develop. Biol, vol. 20, no. 8, pp. 894–902, Oct. 2009, doi: 10.1016/j.semcdb.2009.07.004. 19660567

[15] Gertych A, Ma Z, Tajbakhsh J, Velásquez-Vacca A, and Knudsen BS, “Rapid 3-D delineation of cell nuclei for high-content screening platforms,” Comput. Biol. Med, vol. 69, pp. 328–338, Feb. 2016, doi: 10.1016/j.compbiomed.2015.04.025. 25982066 PMC4440328

[16] Su H, Xing F, Lee JD, Peterson CA, and Yang L, “Automatic myonuclear detection in isolated single muscle fibers using robust ellipse fittingandsparserepresentation,”IEEE/ACMTrans.Comput.Biol.Bioinf, vol. 11, no. 4, pp. 714–726, Jul. 2014, doi: 10.1109/TCBB.2013.151. PMC4669954 26356342

[17] Ronneberger O, Fischer P, and Brox T, “U-Net: Convolutional networks for biomedical image segmentation,” in Medical Image Computing and Computer-Assisted Intervention—MICCAI, Cham, Switzerland: Springer, 2015, pp. 234–241, doi: 10.1007/978-3-319-24574-4_28.

[18] Falk T. , “U-Net: Deep learning for cell counting, detection, and morphometry,” Nature Methods, vol. 16, no. 1, p. 67, 2018. 30559429 10.1038/s41592-018-0261-2

[19] Guerrero-Pena FA, Marrero Fernandez PD, Ren TI, Yui M, Rothenberg E, and Cunha A, “Multiclass weighted loss for instance segmentation of cluttered cells,” in Proc. 25th IEEE Int. Conf. Image Process. (ICIP), Oct. 2018, pp. 2451–2455, doi: 10.1109/ICIP.2018.8451187.

[20] Caicedo JC, Roth J, Goodman A, Becker T, Karhohs KW, Broisin M, Molnar C, McQuin C, Singh S, Theis FJ, and Carpenter AE, “Evaluation of deep learning strategies for nucleus segmentation in fluorescence images,” Cytometry A, vol. 95, no. 9, pp. 952–965, Sep. 2019, doi: 10.1002/cyto.a.23863. 31313519 PMC6771982

[21] Hirsch P. and Kainmueller D, “An auxiliary task for learning nuclei segmentation in 3D microscopy images,” in Proc. 3rd Conf. Med. Imag. Deep Learn., vol. 121, 2020, pp. 304–321.

[22] Vuola AO, Akram SU, and Kannala J, “Mask-RCNN and U-Net ensembled for nuclei segmentation,” in Proc. IEEE 16th Int. Symp. Biomed. Imag. (ISBI), Apr. 2019, pp. 208–212, doi: 10.1109/ISBI.2019.8759574.

[23] Xu Y, Wu T, Gao F, Charlton JR, and Bennett KM, “Improved small blob detection in 3D images using jointly constrained deep learning and hessian analysis,” Sci. Rep, vol. 10, no. 1, pp. 1–12, Jan. 2020, doi: 10.1038/s41598-019-57223-y. 31941994 PMC6962386

[24] Chen H, Qi X, Yu L, Dou Q, Qin J, and Heng P-A, “DCAN: Deep contour-aware networks for object instance segmentation from histology images,” Med. Image Anal, vol. 36, pp. 135–146, Feb. 2017, doi: 10.1016/j.media.2016.11.004. 27898306

[25] Dunn KW, Fu C, Ho DJ, Lee S, Han S, Salama P, and Delp EJ, “DeepSynth: Three-dimensional nuclear segmentation of biological images using neural networks trained with synthetic data,” Sci. Rep, vol. 9, no. 1, Dec. 2019, Art. no. 018295, doi: 10.1038/s41598-019-54244-5. PMC6892824 31797882

[26] Milletari F, Navab N, and Ahmadi S-A, “V-Net: Fully convolutional neural networks for volumetric medical image segmentation,” in Proc. 4th Int. Conf. 3D Vis. (3DV), Stanford, CA, USA, Oct. 2016, pp. 565–571, doi: 10.1109/3DV.2016.79.

[27] Chang C-S, Ding J-J, Chen P-H, Wu Y-F, and Lin S-J, “3-D cell segmentation by improved V-Net architecture using edge and boundary labels,” in Proc. IEEE 2nd Int. Conf. Inf. Commun. Signal Process. (ICICSP), Sep. 2019, pp. 435–439, doi: 10.1109/ICICSP48821.2019.8958531.

[28] Szegedy C, Toshev A, and Erhan D, “Deep neural net-works for object detection,” in Proc. 26th Int. Conf. Neural Inf. Process. Syst. (NIPS), vol. 2, Burges CJC, Bot-Tou L, Ghahramani Z, and Weinberger KQ, Eds. Dec. 2013, pp. 2553–2561.

[29] Sun Y, Wang X, and Tang X, “Deep convolutional network cascade for facial point detection,” in Proc. IEEE Conf. Comput. Vis. Pattern Recognit., Portland, OR, USA, Jun. 2013, pp. 3476–3483, doi: 10.1109/CVPR.2013.446.

[30] Kainz P, Urschler M, Schulter S, Wohlhart P, and Lepetit V, “You should use regression to detect cells,” in Medical Image Computing and Computer-Assisted Intervention—MICCAI, 2015, Cham, Switzerland: Springer, 2015, pp. 276–283, doi: 10.1007/978-3-319-24574-4_33.

[31] Tan R, Zhang J, Chen P, Wang B, and Xia Y, “Cells Counting with Convolutional Neural Network,” in Intelligent Computing Methodologies, Cham, Switzerland: Springer, 2018, pp. 102–111, doi: 10.1007/978-3-31995957-3_12.

[32] Sirinukunwattana K, Raza SEA, Tsang Y-W, Snead DRJ, Cree IA, and Rajpoot NM, “Locality sensitive deep learning for detection and classification of nuclei in routine colon cancer histology images,” IEEE Trans. Med. Imag, vol. 35, no. 5, pp. 1196–1206, May 2016, doi: 10.1109/TMI.2016.2525803. 26863654

[33] Xie W, Noble JA, and Zisserman A, “Microscopy cell counting and detection with fully convolutional regression networks,” Comput. Methods Biomech. Biomed. Eng., Imag. Visualizat, vol. 6, no. 3, pp. 283–292, May 2018, doi: 10.1080/21681163.2016.1149104.

[34] Xie Y, Xing F, Shi X, Kong X, Su H, and Yang L, “Efficient and robust cell detection: A structured regression approach,” Med. Image Anal, vol. 44, pp. 245–254, Feb. 2018, doi: 10.1016/j.media.2017.07.003. 28797548 PMC6051760

[35] Höfener H, Homeyer A, Weiss N, Molin J, Lundström CF, and Hahn HK, “Deep learning nuclei detection: A simple approach can deliver state-of-the-art results,” Computerized Med. Imag. Graph, vol. 70, pp. 43–52, Dec. 2018, doi: 10.1016/j.compmedimag.2018.08.010. 30286333

[36] Tokuoka Y, Yamada TG, Mashiko D, Ikeda Z, Hiroi NF, Kobayashi TJ, Yamagata K, and Funahashi A, “3D convolutional neural networks-based segmentation to acquire quantitative criteria of the nucleus during mouse embryogenesis,” NPJ Syst. Biol. Appl, vol. 6, no. 1, pp. 1–12, Oct. 2020, doi: 10.1038/s41540-020-00152-8. 33082352 PMC7575569

[37] Ho DJ, Montserrat DM, Fu C, Salama P, Dunn KW, and Delp EJ, “Sphere estimation network: Three-dimensional nuclei detection of fluorescence microscopy images,” J. Med. Imag, vol. 7, no. 04, Aug. 2020, Art. no. 044003, doi: 10.1117/1.JMI.7.4.044003. PMC7451995 32904135

[38] Ram S, Nguyen VT, Limesand KH, and Rodriguez JJ, “Combined detection and segmentation of cell nuclei in microscopy images using deep learning,” in Proc. IEEE Southwest Symp. Image Anal. Interpretation (SSIAI), Mar. 2020, pp. 26–29, doi: 10.1109/SSIAI49293.2020.9094614.

[39] Sadanandan SK, Ranefall P, Guyader SL, and Wählby C, “Automated training of deep convolutional neural networks for cell segmentation,” Sci. Rep, vol. 7, no. 1, pp. 1–7, Aug. 2017, doi: 10.1038/s41598-017-07599-6. 28798336 PMC5552800

[40] Otsuka K. 3-D Deep Learning?: Lung Tumor Segmentation. MATLAB Central File Exchange. Accessed: Feb. 26, 2021. [Online]. Available: https://www.mathworks.com/matlabcentral/fileexchange/71521-3-ddeep-learning-lung-tumor-segmentation

[41] Glorot X. and Bengio Y, “Understanding the difficulty of training deep feedforward neural networks,” in Proc. 13th Int. Conf. Artif. Intell. Statist., Mar. 2010, pp. 249–256. Accessed: Jan. 27, 2021. [Online]. Available: http://proceedings.mlr.press/v9/glorot10a.html

[42] Liu Y, Jenkins MW, Watanabe M, and Rollins AM, “A simple optical clearing method for investigating molecular distribution in intact embryonic tissues (conference presentation),” Proc. SPIE, vol. 10472, Mar. 2018, Art. no. 104720P, doi: 10.1117/12.2291193.

[43] Yushkevich PA, Piven J, Hazlett HC, Smith RG, Ho S, Gee JC, and Gerig G, “User-guided 3D active contour segmentation of anatomical structures: Significantly improved efficiency and reliability,” NeuroImage, vol. 31, no. 3, pp. 1116–1128, Jul. 2006, doi: 10.1016/j.neuroimage.2006.01.015. 16545965

[44] Kingma DP and Ba J, “Adam: A method for stochastic optimization,” Jan. 2014, arXiv:1412.6980. Accessed: Feb. 15, 2021. [Online]. Available: http://arxiv.org/abs/1412.6980

[45] Baddeley A, Rubak E, and Turner R, Spatial Point Patterns: Methodology and Applications with R. Boca Raton, FL, USA: CRC Press, 2015.

[46] Schindelin J, Arganda-Carreras I, Frise E, Kaynig V, Longair M, Pietzsch T, Preibisch S, Rueden C, Saalfeld S, Schmid B, Tinevez J-Y, White DJ, Hartenstein V, Eliceiri K, Tomancak P, and Cardona A, “Fiji: An open-source platform for biological-image analysis,” Nature Methods, vol. 9, no. 7, pp. 676–682, Jul. 2012, doi: 10.1038/nmeth.2019. 22743772 PMC3855844

[47] Schindelin J, Rueden CT, Hiner MC, and Eliceiri KW, “The ImageJ ecosystem: An open platform for biomedical image analysis,” Mol. Reprod. Develop, vol. 82, nos. 7–8, pp. 518–529, Jul. 2015, doi: 10.1002/mrd.22489. PMC5428984 26153368

