# Supplementary material for: Nuclei Detection for 3D Microscopy With a Fully Convolutional Regression Network
Source: IEEE Access. Author manuscript; Available in PMC 2022 Jan 11. (PMC8751907; doi:10.1109/ACCESS.2021.3073894)
Supplement: access-3073894-mm [file NIHMS1697028-supplement-access-3073894-mm.zip › access-3073894-mm/readme.pdf]

Description: The first video shows three-dimensional fly-through nuclei centroids identified using VRegNet in a quail heart at embryonic day 9 (heart #1), true positives (cyan), false negatives (magenta), and false positives (yellow). The second video shows three-dimensional fly-through showing nuclei centroids identified using Vnet-3W in a quail heart at embryonic day 9 (heart #1), true positives (cyan), false negatives (magenta), and false positives (yellow). The third video shows three-dimensional fly-through showing nuclei centroids identified using Vnet-2W in a quail heart at embryonic day 9 (heart #1). True positives (cyan), false negatives (magenta), and false positives (yellow).

Size: 25.1 MB

Packing List: access-3073894-mm.zip

Player Information: any .avi player

Contact Information:

Dr. Maryse Lapierre-Landry

Case Western Reserve Univ., Dept. of Biomedical Engineering

Cleveland, OH, USA 44106

Email: mxl1010@case.edu
